# Case report: Liquid biopsy, the sooner the better?

**DOI:** 10.3389/fonc.2022.1089108

**Published:** 2022-12-15

**Authors:** Quentin Dominique Thomas, Julien Colard-Thomas, Delphine Delansay, Fanny Leenhardt, Jerome Solassol, Julie A. Vendrell, Xavier Quantin

**Affiliations:** ^1^ Department of Medical Oncology, Montpellier Cancer Institute (ICM), Montpellier, France; ^2^ Oncogenic Pathways in Lung Cancer, Montpellier Cancer Research Institute (IRCM), University of Montpellier (UM), Montpellier, France; ^3^ Pharmacy department, Montpellier Cancer institute (ICM), Montpellier, France; ^4^ Department of Pathology, Montpellier University Hospital (CHU) Montpellier, Arnaud de Villeneuve Hospital, Montpellier, France

**Keywords:** lung cancer, ctDNA = circulating tumor DNA, liquid biopsy, EGFR, thrombotic microangiopathy (TMA), osimertinib

## Abstract

The detection of circulating tumor DNA (ctDNA) by liquid biopsy is taking an increasing role in thoracic oncology management due to its predictive and prognostic value. For non-small cell lung cancer, it allows the detection of molecular mutations that can be targeted with tyrosine kinase inhibitors (TKIs). We report the case of a patient with life-threatening hepatocellular failure and thrombotic microangiopathy at the diagnosis. A salvage chemotherapy was attempted, resulting in a major worsening of her general condition and the decision to stop all anti-cancer treatment. The liquid biopsy performed at the time of immunohistochemical non-small cell lung cancer diagnosis revealed within 7 days the presence of an epidermal growth factor receptor (*EGFR)* ^DEL19^ activating mutation with 736,400 DNA copies/ml of plasma. It was finally decided to attempt a treatment with osimertinib (third generation anti-EGFR TKI) despite the fact that the patient was in a pre-mortem situation. Osimertinib led to a significant and prompt improvement of her performance status after only one week of treatment. The tumor tissue genotyping performed by next-generation sequencing (NGS) was available 10 days after starting TKI treatment. It revealed in addition to the *EGFR* ^DEL19^ mutation, a *JAK3* and *EGFR* amplification, highlighting the complex interactions between EGFR and the JAK/STAT signaling pathways. The first CT-scan performed after 2 months under osimertinib showed a tumor morphologic partial response. The biological assay showed a major decrease in the *EGFR* ^DEL19^ mutation ctDNA levels (40.0 copies/ml). The liquid biopsy allowed an early implementation of a targeted therapy without which the patient would probably be dead. Testing for ctDNA should be discussed routinely at diagnosis in addition to tumor tissue genotyping for patient with metastatic non-small cell lung cancer that raise the clinical profile of oncogenic addiction.

## Introduction

Oncogenic activating mutations are common for patients with a non-small cell lung cancer (NSCLC) benefiting from treatment by targeted therapies (1). Systematic molecular and biomarker analysis is performed at diagnosis. It is recommended, when feasible, that testing be carried out *via* a broad, panel-based approach, next-generation sequencing (NGS) in clinical laboratories. Testing failure often occurs due to the small size of tissue samples obtain *via* mini-invasive technics. A second clinical limitation is the delay in the delivery of molecular results, which in most cases takes around three weeks. In most clinical situations this delay is acceptable for optimal therapeutic decision making. However, in some clinical presentations where patients are life-threatening at diagnosis, a technology that allows a faster molecular testing may be necessary. Liquid biopsy offers a solution to these both challenges allowing results for circulating tumor DNA (ctDNA) within approximately one week. Detection of ctDNA guides therapy decisions and has recently been shown, in a large prospective cohort of 1,127 patients with NSCLC, to be associated with shorter survival (hazard ratio (HR), 2.05; 95% confidence interval (CI), 1.74-2.42; P < 0.001) independently of clinicopathologic features and metabolic tumor volume in a large prospective cohort of 1,127 patients with NSCLC (2).

Epidermal growth factor receptor (*EGFR*) activating mutations, such as exon 19 deletion (*EGFR* ^DEL19^) or the *EGFR* ^L858R^ mutation in exon 21, occur in 10-15% of Caucasian patients with NSCLC. For these patients with metastatic disease, specific tyrosine kinase inhibitors (TKIs) are recommended. Osimertinib, a third-generation *EGFR* TKI, has become the first-line standard of care for patients with *EGFR*-mutated NSCLC (3). We report the case of a patient with a life-threatening medical situation at diagnosis of NSCLC with severe hepatocellular failure and thrombotic microangiopathy for which liquid biopsy revealed an *EGFR* activating alteration. The detection of this mutation allowed us to rapidly initiate treatment with *EGFR* TKI, without which the patient would have died from complications of her cancer at diagnosis.

## Case report

A 61-year-old female patient with no personal medical history except osteoporosis and no familial history of cancer; former smoker estimated at 2 packs/year presented in May 2022 severe and rapid deterioration in general condition resulting in severe asthenia associated with complete anorexia, nausea and epigastric pain. Clinical examination revealed a painful hepatomegaly without other clinical abnormality, and hepatic sonogram showed diffuse bilobar lesions.

On June 7, a computerized tomography (CT) scan showed diffuse hepatic lesions associated with multiple sus and sub-diaphragmatic lymphadenopathies, pleural effusion, diffuse bone and pulmonary lesions.

On June 15, a liver biopsy led to the diagnosis of a CK7+; CK20-; TTF1+; SATB2-; GATA 3- mucus-secreting carcinoma leading to the diagnosis of T1aN3M1c bronchopulmonary adenocarcinoma according to the 8th version of the TNM classification. Blood tests on June 17 revealed anemia with hemoglobin at 7.5 g/dL; international normalized ratio (INR) increase at 1.51; hepatic enzymes increase with gamma-glutamyltransferase at 458 U/L; alkaline phosphatase at 703 U/L; aspartate aminotransferase at 156 U/L; alanine aminotransferase at 129 U/L; total and conjugated bilirubin increase at respectively 56.9 µmol/L and 32.6 µmol/L. The suspicion of acute liver failure led to her hospitalization. Eastern Cooperative Oncology Group Performance Status (ECOG PS) was 3, oxygen was needed at 1-2 L/min. Viral serologies were negatives (Hepatitis B, Hepatitis C, HIV, Cytomegalovirus), there were no history of alcoholic intoxication or recent introduction of new medication explaining the hepatic perturbations. Treatment was initially postponed waiting for the results of tumor molecular biology, considering the high probability of an oncogenic driver for this patient former smoker under 5 packs/year. A liquid biopsy was realized on June 21 to research ctDNA alterations.

On June 22, following to appearance of encephalopathy due to hepatic failure it was decided to initiate salvage chemotherapy with weekly paclitaxel/carboplatin regimen. After 48 hours, the patient developed a massive hematoma in front of central venous catheter associated with clinical degradation and increase of oxygen need to 4 L/min. Blood tests revealed grade 3 according to common terminology criteria for adverse events v5.0 (CTCAE v5.0) platelet count decrease leading to an initial diagnosis of disseminated intravascular coagulation. Subsequent analysis revealed anemia, negative haptoglobin, and schizocytes on blood smear, rectifying the diagnosis of paraneoplastic thrombotic microangiopathy. A new CT-scan showed massive pleural and peritoneal effusion. This dramatic clinical and biological deterioration resulted in stopping antitumor treatment and focusing on best supportive care.

**Figure 1:**
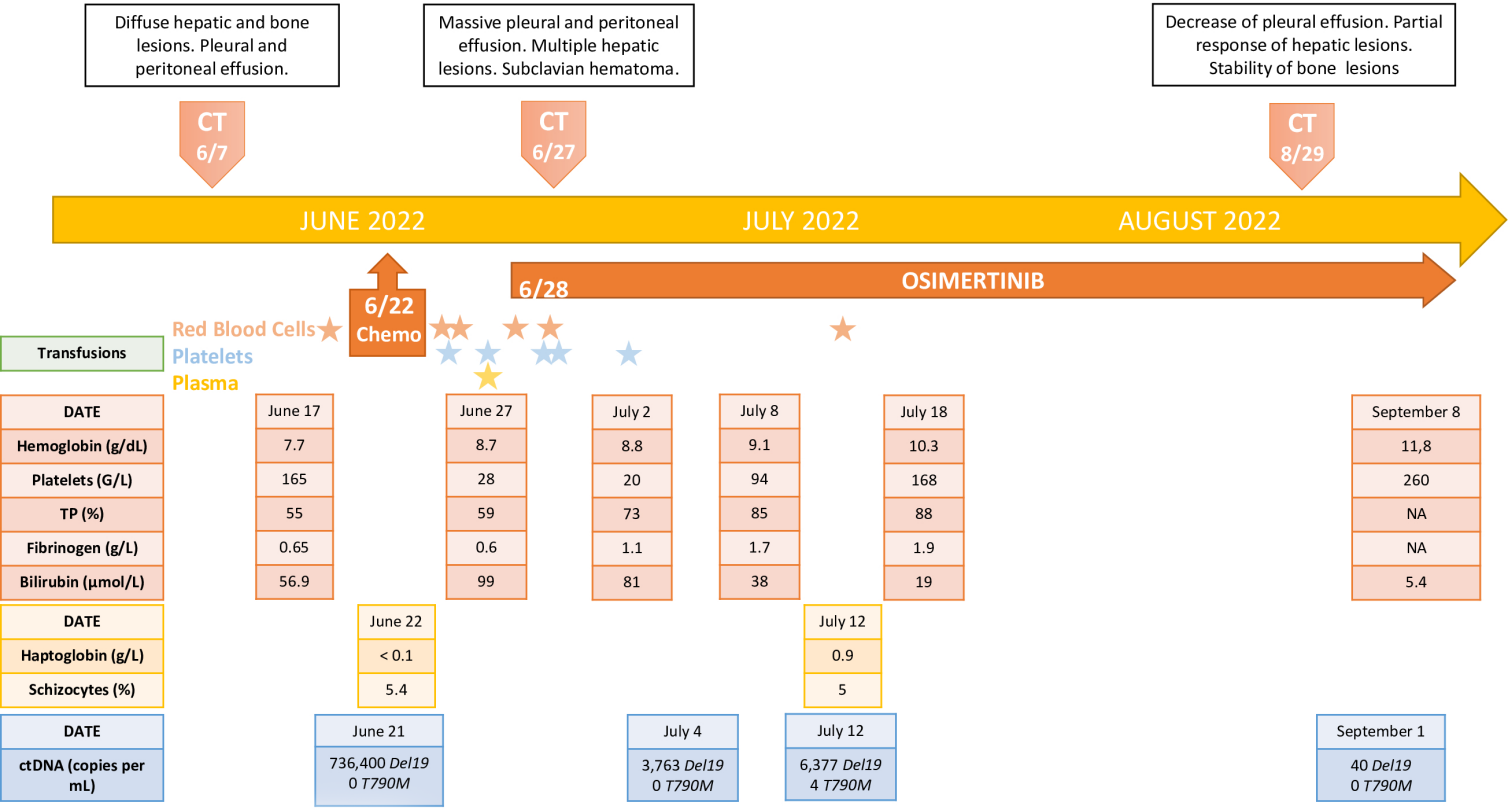
Timeline of the disease course.

However, on June 27, the results of ctDNA performed on June 21 by digital droplet PCR (ddPCR) revealed the presence of an *EGFR* ^DEL19^ mutation with very high amount of copies (736,400 per mL of plasma). A last-ditch attempt was decided by initiating a TKI treatment with osimertinib 80 mg per day, first dose received on June 28 (**Figure 1**).

After one week of treatment, a clinical and biological improvement was observed with decrease of oxygen requirement, edemas, lactate dehydrogenase and INR. On July 4, a control of ctDNA showed a drastic drop to 3,763 copies of mutated DNA/mL of plasma. A second control was performed on July 12, finding 6,377 copies/mL for *EGFR* ^DEL19^ and a possible *EGFR* ^T790M^ resistance mutation at 4.0 copies/mL (**Figure 1**).

Clinically, oxygen was stopped on July 8, after 10 days of treatment with osimertinib. Molecular alterations were also investigated on the hepatic punctured tissue: the results came out on July 7. The NGS performed on tissue biopsy confirmed the presence of an *EGFR* ^DEL 19^ with a variant allele frequency of 88.45%. The NGS experiment also revealed the presence of an *EGFR* amplification with 10 copies of genes and a Janus Kinase 3 (*JAK3*) amplification with 14 copies of genes.

**Figure 2:**
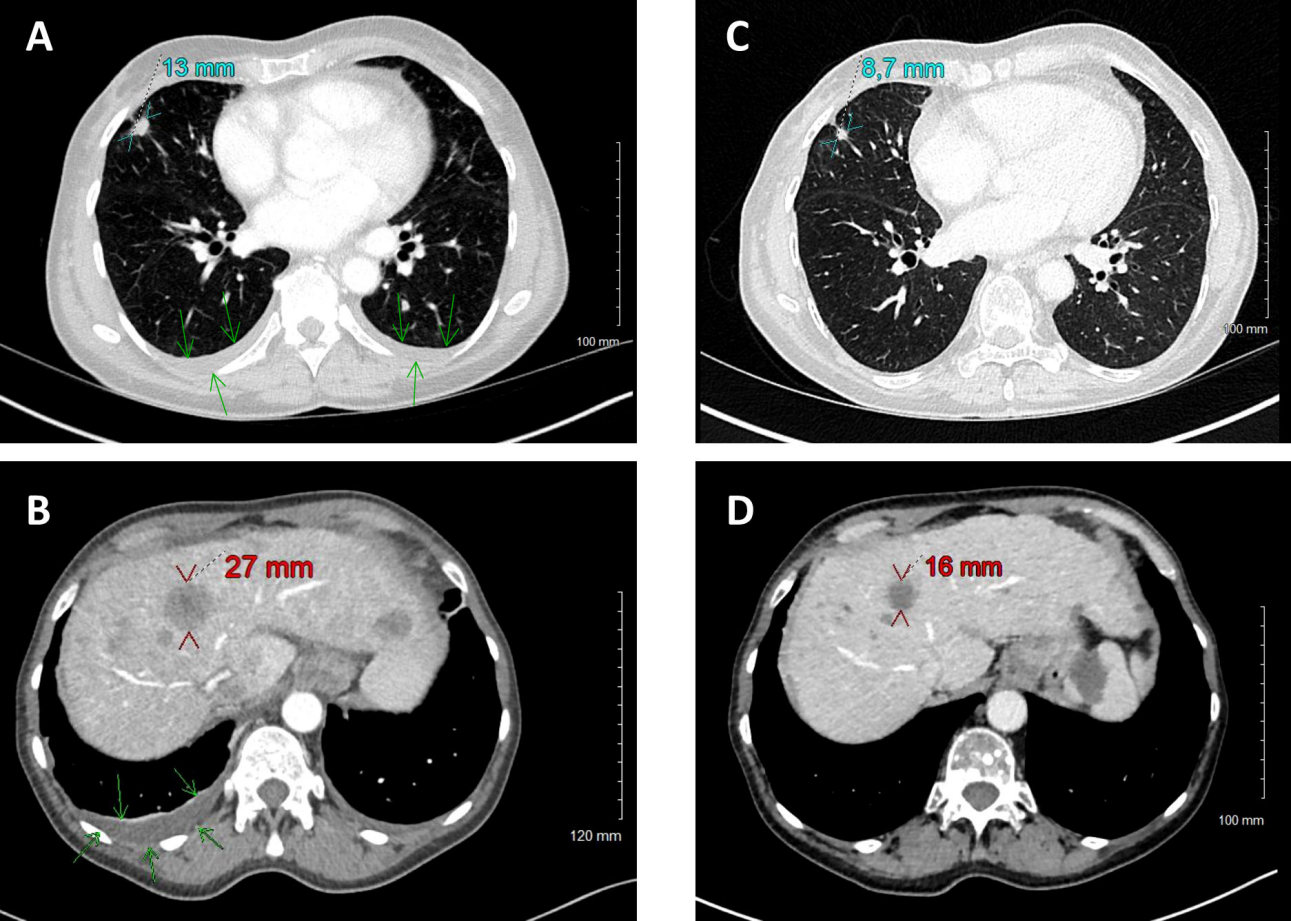
Comparison of CT scans before (June 7, **A**, **B**) and with osimertinib treatment (August 29, **C**, **D**). Size reduction of primary tumor (cyan) and target hepatic metastasis (red). Disparition of pleural effusion (green arrows).

After three weeks of treatment by osimertinib associated with supportive care, the patient was able to return directly to her home without requiring a rehabilitation. After 2 months of treatment with osimertinib, the patient was clinically asymptomatic and has no side effects from the TKI. The CT-scan performed shows a partial morphological response according to RECIST1.1 criteria (**Figure 2**). Control of ctDNA rate revealed a residual value for the *EGFR* ^DEL19^ activating mutation (40.0 copies/mL) and the disappearance of the *EGFR* ^T790M^ mutation (**Figure 1**).

To date, after 5 months of full-dose treatment with osimertinib 80mg/day, the patient still has an excellent tolerance of the treatment with maintained efficacy.

## Discussion

To the best of our knowledge, we present the case of a NSCLC EGFR-mutated patients with one of the highest levels of ctDNA reported in the literature (4). This patient who was ECOG PS 4 and in terminal phase of hepatocellular insufficiency and thrombotic microangiopathy improved clinically in 10 days (ECOG PS0) with a normalization of her biological results in 15 days under osimertinib. Clinical practice guidelines recommend treating patients with ECOG PS 3-4 with best supportive care. Patients with NSCLC with oncogenic addiction represent a particular population for which targeted therapies can be proposed even for ECOG PS 3-4 (5). This case report highlights the possibility of clinical improvement under TKI even in life-threatening situations. Several case series reports support this observation, showing that an invasive attitude can be beneficial for these patients. Indeed, patients intubated in intensive care units due to respiratory failure related to the disease or patients who require enteral feeding to administer TKI tablets benefit from the treatment with a clear recovery of their quality of life and a clear improvement of their survival (6) (7).

From a molecular point of view, this patient presents several characteristics that deserve to be discussed. In our center, we systematically search for gene copy number variation by NGS amplification technique performed on tumor tissue. This analysis identified in addition to the *EGFR* ^DEL 19^ mutation, an amplification of the *EGFR* gene as well as an amplification of the *JAK3* gene. *EGFR* amplification has been described identified as a potential mechanism of primary resistance to third generation-TKIs resistance (8). In addition, *EGFR* mutations can induce abnormal activation of the downstream JAK/STAT signaling pathway considered as one of the central communication nodes in the cell function. The JAK family consists of non-receptor tyrosine protein kinases with four main members (JAK 1-3 and TYK2). Dysregulation of the JAK/STAT pathway promote abnormal tumor proliferation, angiogenesis, invasion and metastasis (9). There are actually several clinical trials evaluating the role of JAK/STAT pathway inhibitors in NSCLC *EGFR*-mutated (**Table 1**).

**Table 1:** Published and ongoing trial evaluating inhibitors of JAK/STAT pathway for EGFR-mutated non-small cell lung cancer.

Drug regimens	ClinicalTrial.gov	Therapeutic line	Development stage	Patient included	Clinical outcomes
AZD4205 (JAK 1 inhibitor) + Osimertinib	NCT03450330	Activating EGFR mutation positive NSCLC and have failed prior EGFR TKIs treatment	Phase I/II Completed	10	Safety and tolerability
Ruxolitinib (JAK1 + JAK2 inhibitor) + Afatinib	NCT02145637	Disease progression after platinum doublet (all), EGFR TKI (if EGFR mutant), and crizotinib (if ALK positive)	Phase I Completed	30	- ORR: 23.3% (7PR/0CR) - DCR: 93.3% - PFS: 4.9months (95% CI, 2.4-7.5)
Itacitinib (JAK 1 inhibitor) + Osimertinib	NCT02917993	Progressed on or after treatment with an EGFR tyrosine kinase inhibitor (TKI). Additional lines of systemic therapy including investigational agents for locally advanced or metastatic NSCLC are allowed.	Phase I/II Active not recruting	59	Safety and tolerability ORR
Momelotinib (JAK1/2 and TANK-binding kinase 1 (TBK1) inhibitor) + Erlotinib	NCT02206763	Patients with EGFR TKI-naïve NSCLC	Phase I Completed	11	-ORR: 54.5% (90% CI 27.1-80.0) -PFS : 9.2 months (90% CI 6.2-12.4)
AZD1480 (JAK 2 inhibitor)	NCT01219543	Asian patients with advanced EGFR or ROS mutant NSCLC	Phase I Completed	47	Safety and tolerability

DCR, disease control rate; EGFR, Epidermal growth factor receptor; NSCLC, non-small cell lung cancer; JAK, Janus kinase; ORR, Objective response rate; PFS, Progression free survival; TKI, Tyrosine kinase inhibitor.

Regarding ctDNA, complete clearance of the activating mutation under anti-*EGFR* TKI treatment has proven prognostic value. It is also a powerful tool for anticipating tumor relapse (10). Concomitant tissue detection of preexisting *EGFR* ^T790M^ associated with *EGFR* activating mutations is an event occurring in 1-5% of *EGFR*-NSCLC, conferring a worse prognosis value (11) (12). Liquid biopsy detection of a pre-existing *EGFR* ^T790M^ mutation by ddPCR also appears to have prognostic value in the management of NSCLC patients (13). In our case, the detection of an *EGFR* ^T790M^ mutation is probably a background noise inherent to the ddPCR technique. Indeed, the appearance of an *EGFR* ^T790M^ mutation is not observed under osimertinib, whose mechanism of action precisely targets this mutation. This hypothesis is confirmed by the absence of detection of *EGFR* ^T790M^ mutation after 2 months of treatment with osimertinib and the continued decrease in the ctDNA rate of the *EGFR* ^DEL19^ mutation.

Interestingly, the number of copies of mutated ctDNA detected increased during the course of treatment by osimertinib and finally decreased secondarily. This observation led us to suspect an early resistance to osimertinib. The kinetics of the decrease of the ctDNA level after 2 months of treatment show us that this rebound of the ctDNA level is probably related to the loss of effectiveness of the chemotherapy carried out in first intention by paclitaxel/carboplatin regimen. Indeed, the chemotherapy probably had an anti-tumor activity associated with the treatment with osimertinib which contributed to the decrease of the *EGFR* ^DEL19^ ctDNA level. This observation reinforces the importance of clinical trials combining chemotherapy with TKIs for NSCLC with oncogenic addiction (NCT04035486; NCT05200481).

Advantages and disadvantages of liquid biopsy in comparison to tissue biopsy for tumor genotyping in advanced or metastatic NSCLC can be summarized as follows: liquid biopsy present high concordance rate with tissue biopsy; the technique is faster repeatable over time and minimally invasive. Liquid biopsy better capture tumor heterogeneity and clonal evolution under treatment. However, liquid biopsy is less sensitive, does not allow the evaluation of non-DNA biomarkers, in particular the programmed death ligand-1 (PD-L1) status, which is fundamental in the decision of the therapeutic strategy in first metastatic line. It also increases costs if performed concurrently with tissue testing (14). Some complementary approaches of liquid biopsy including characterization of circulating tumor cells and tumor mutation burden may be a promising tool to help clinicians in therapeutic decision-making for advanced NSCLC (15). Liquid biopsy is an increasingly important tool in oncology. It provides prognostic information for curative patients (16). It is already a leading tool in the stratification of the therapeutic strategy for adjuvant chemotherapy for colorectal patients (17). One of the main challenges for liquid biopsy research in the coming years will be to optimize its use for the detection of early-stage neoplasms or tumor relapse by detecting a minimal residual disease. In this context, efforts are being made to improve the detection of smallest amounts of ctDNA in the “sea” of normal circulating free DNA. Ultrasensitives liquid biopsy technologies will probably be a prerequisite to implement liquid biopsy in these strategies (18).

We report here a rare situation of the added value of liquid biopsy. It provides faster information on molecular tumor alterations than tissue biopsy. This time saving can lead to propose earlier targeted therapy to NSCLC patients. Our opinion is that a liquid biopsy must be discussed routinely at the diagnosis of NSCLC in a complementary approach with tumor tissue genotyping.

## Data availability statement

The original contributions presented in the study are included in the article/Supplementary Material. Further inquiries can be directed to the corresponding author.

## Ethics statement

Written informed consent was obtained from the individual (s) for the publication of any potentially identifiable images or data included in this article.

## Author contributions

QT: Conceptualization; Writing - Original Draft; Writing - Review & Editing; Visualization. JC-T: Writing - Original Draft; Writing - Review & Editing; Visualization. DD, FL, JS, JV, and XQ: Writing - Review & Editing; Supervision. All authors contributed to the article and approved the submitted version.
